# Enhancement of ZnO@RuO_2_ bifunctional photo-electro catalytic activity toward water splitting

**DOI:** 10.3389/fchem.2023.1173910

**Published:** 2023-04-27

**Authors:** Katarina Aleksić, Ivana Stojković Simatović, Ana Stanković, Ljiljana Veselinović, Stevan Stojadinović, Vladislav Rac, Nadežda Radmilović, Vladimir Rajić, Srečo Davor Škapin, Lidija Mančić, Smilja Marković

**Affiliations:** ^1^ Institute of Technical Sciences of SASA, Belgrade, Serbia; ^2^ Faculty of Physical Chemistry, University of Belgrade, Belgrade, Serbia; ^3^ Faculty of Physics, University of Belgrade, Belgrade, Serbia; ^4^ Faculty of Agriculture, University of Belgrade, Zemun, Serbia; ^5^ Vinča Institute of Nuclear Sciences, University of Belgrade, Belgrade, Serbia; ^6^ Jožef Stefan Institute, Ljubljana, Slovenia

**Keywords:** ZnO, RuO_2_, composites, water splitting, photo-electro catalysis

## Abstract

Catalytic materials are the greatest challenge for the commercial application of water electrolysis (WEs) and fuel cells (FCs) as clean energy technologies. There is a need to find an alternative to expensive and unavailable platinum group metal (PGM) catalysts. This study aimed to reduce the cost of PGM materials by replacing Ru with RuO_2_ and lowering the amount of RuO_2_ by adding abundant and multifunctional ZnO. A ZnO@RuO_2_ composite in a 10:1 molar ratio was synthesized by microwave processing of a precipitate as a green, low-cost, and fast method, and then annealed at 300°C and 600°C to improve the catalytic properties. The physicochemical properties of the ZnO@RuO_2_ composites were investigated by X-ray powder diffraction (XRD), Raman and Fourier transform infrared (FTIR) spectroscopy, field emission scanning electron microscopy (FESEM), UV-Vis diffuse reflectance spectroscopy (DRS), and photoluminescence (PL) spectroscopy. The electrochemical activity of the samples was investigated by linear sweep voltammetry in acidic and alkaline electrolytes. We observed good bifunctional catalytic activity of the ZnO@RuO_2_ composites toward HER and OER in both electrolytes. The improved bifunctional catalytic activity of the ZnO@RuO_2_ composite by annealing was discussed and attributed to the reduced number of bulk oxygen vacancies and the increased number of established heterojunctions.

## 1 Introduction

The simultaneous rapid decline of fossil fuels as energy resources and their deteriorating effects due to environmental pollution have inspired scientists and engineers worldwide to develop clean energy through renewable energy sources (Li et al., 2019; Devadas et al., 2020). Water electrolysis, regenerative fuel cells, and rechargeable metal–air batteries are among the sustainable energy conversion systems able to convert molecules abundant in the environment, such as water, carbon dioxide, and nitrogen, into useful products such as hydrogen, hydrocarbons, and ammonia (Jiang et al., 2019; Li et al., 2019; Ullah et al., 2021; Li et al., 2022). Each of these conversion systems, i.e., water electrolysis, regenerative fuel cells, and rechargeable metal–air batteries, use different electrochemical conversion pathways. More specifically, the hydrogen evolution reaction (HER) and the oxygen evolution reaction (OER) rule the energy storage system through electrocatalytic water splitting; the hydrogen oxidation reaction (HOR) and the oxygen reduction reaction (ORR) control fuel cells; and the ORR and OER are predominant in electrochemically rechargeable metal–air batteries (Li et al., 2019). Due to the slow kinetics, the main problem in the electrochemical reactions, with a large overpotential and low round-trip efficiency as consequences, the development and implementation of highly efficient electrocatalysts are needed for HER, OER, and ORR to increase the reaction rate and the efficiency of the overall system (Jiang et al., 2019; Li et al., 2019; Jin et al., 2022). A plethora of developed electrocatalysts can be used to improve individual electrocatalytic reactions. However, reducing the manufacturing and operating costs of the devices requires the development of electrocatalysts that efficiently combine reactions such as HER/OER, ORR/OER, and HOR/ORR. Since HER, OER, and ORR occur in completely different potential windows, few commercially available catalysts can act as multifunctional electrocatalysts; more specifically, the best OER catalyst usually has poor HER activity, while the best ORR catalyst does not have the best OER catalysis performance, and *vice versa* (Li et al., 2019). Currently, the best electrocatalysts for HER and ORR are Pt-based materials, while RuO_2_ and IrO_2_ exhibit the best OER electrocatalytic activity (Li et al., 2019; Devadas et al., 2020; Xie et al., 2020; Jin et al., 2022; Milikić et al., 2023). However, despite their high catalytic activity toward OER and their potential for use as efficient bifunctional HER/OER catalysts, both RuO_2_ and IrO_2_ are limited by their poor long-term stability, natural scarcity, and high price (Guan et al., 2018; Liang et al., 2020). Therefore, interest is increasing in the development of low-cost and more stable efficient bi- or even trifunctional catalysts. So far, various composites with precious metal oxides such as RuO_2_ and IrO_2_, and abundant materials, such as ZnO, MoO_3_, WO_3_, and g-C_3_N_4_, have been developed and tested as catalysts (Tariq et al., 2020; Wang et al., 2021a; Jin et al., 2022; Nur et al., 2022).

Due to their high electron mobility and transfer efficiency (115–155 cm^2^V^−1^s^−1^), intrinsic stability, non-toxicity, and environmental compatibility, zinc oxide (ZnO)-based materials offer great opportunities as catalysts. ZnO is an attractive photo-electro catalyst due to its wide-band gap energy at room temperature (3.37 eV), which can be tuned by metal and non-metal ion doping, incorporation of crystalline defects in the form of vacancies and interstitials, modification of particle morphology and surface chemistry, etc. (Chen et al., 2011; Li et al., 2014; Xia et al., 2014; Marković et al., 2016; Marković et al., 2017; Marković et al., 2019; Rajić et al., 2020).

We previously demonstrated the promise of ZnO-based nanostructured materials as photo-electro catalysts for OER and bifunctional catalysis for OER/ORR in moderately alkaline solutions (Marković et al., 2019; Rajić et al., 2020). The present study aimed to additionally improve the optical and catalytic properties of ZnO particles through composites with commercially available RuO_2_ particles. *In situ* precipitation of Zn(OH)_2_ onto RuO_2_ and microwave processing of the precipitate were employed to reduce the number of oxygen vacancies and improve local crystal symmetry. To our knowledge, no other published data have described the same processing of ZnO@RuO_2_ composite particles. In this study, we used ZnO:RuO_2_ in a 10:1 molar ratio. The as-prepared 10ZnO@RuO_2_ composite was additionally annealed at 300°C and 600°C to further reduce the number of bulk oxygen vacancies. We investigated the crystal structure, morphology, and optical and photo-electro catalytic properties of the processed 10ZnO@RuO_2_ catalyst particles in detail. We discussed the possibilities of using ZnO@RuO_2_ composites as photo-electro catalysts for HER, OER, and ORR in both alkaline and acidic solutions.

## 2 Materials and methods

### 2.1 Materials

Zinc chloride (ZnCl_2_, purity >99.5, Lach-Ner, Neratovice, Czech Republic) and ruthenium oxide (RuO_2_, purity >99.9, Sigma-Aldrich) were used as zinc and ruthenium sources, respectively. Sodium hydroxide (NaOH, purity >98%, CARLO ERBA Reagents) was used as a precipitating agent. All chemicals were used as supplied by the manufacturers, without additional purification. Distilled water was used as a solvent and dispersant and to rinse the powder, while absolute ethanol (Zorka, Šabac) was used for the final rinse.

### 2.2 Synthesis of the composite catalysts

A composite of ZnO and RuO_2_ in a 10:1 M ratio was prepared by microwave processing of an *in situ* precipitated Zn(OH)_2_ on RuO_2_. For this purpose, 0.044 g of RuO_2_ was dispersed in 100 mL of distilled water under constant stirring with a magnetic stirrer. After stirring for 5 min, 0.8976 g ZnCl_2_ was added to the RuO_2_-water dispersion. Next, 20 mL of 1.75 M NaOH was added dropwise to the mixture of dispersed RuO_2_ and zinc solution with constant stirring. After stirring at 50°C for 90 min in total, the as-prepared black precipitate was microwave processed in a domestic oven (2.45 GHz, 130 W) for 5 min. After cooling to room temperature, the precipitate was centrifuged at 5,000 rpm for 10 min, rinsed five times with distilled water, and then with absolute ethanol to remove the surface residues of the starting chemical solutions. The synthesized powder was dried in an oven at 80°C for 24 h.

To vary the particle crystallinity and the number of oxygen vacancies, the composite sample was annealed at two different temperatures. The annealing temperatures were selected based on thermogravimetric and differential thermal analysis (TG-DTA) of the as-prepared ZnO@RuO_2_ composite. The analysis was determined by simultaneous TG-DTA (Setsys, SETARAM Instrumentation, Caluire, France) between 30°C and 800°C under an airflow of 20 mL/min in an Al_2_O_3_ pan. The heating profile was as follows: the material was stabilized at 30°C for 5 min and then heated to 800°C at 10°/min. From the TG-DTA, curves 300°C and 600°C were selected as further annealing temperatures. The composite was annealed in a tube furnace in an air atmosphere at a heating rate of 10°/min and a dwell time of 1 h.

This study denotes the prepared photo-electro catalysts as 10ZnO@1RuO_2_, 10ZnO@1RuO_2_-300, and 10ZnO@1RuO_2_-600, where the numbers 10 and 1 signify the molar ratio of ZnO and RuO_2_ and 300 and 600 signify the annealing temperatures.

### 2.3 Characterization

The Fourier transform infrared (FTIR) spectrum was recorded using a Thermo Scientific™ Nicolet™ iS™10 Spectrometer equipped with an attenuated total reflectance (ATR) accessory. The measurements were performed in the wavenumber range of 400–4,000 cm^–1^ with a resolution of 4 cm^–1^. XRD data were collected at room temperature using a Philips PW 1050 diffractometer with CuK_α1,2_ (*λ* = 1.54178 Å) Ni-filtrated radiation. The diffraction intensity was measured from 20° to 80° 2θ, with a step size of 0.05° and a counting time of 5 s per step. The working conditions were 40 kV and 20 mA. The crystal phases were identified using Mach!3 database software (Putz and Brandenburg, 2023), with reference to the Crystallography Open Database (COD) patterns (Crystallography Open Database, 2023). The unit cell parameters were calculated using LSUCRI software and the least-squares method (Garvey, 1986), while crystallite sizes (*D*) were calculated from XRD line-broadening using the Scherrer equation (Klug and Alexander, 1954). The μ-Raman spectra were recorded at room temperature in the frequency range of 50–1,000 cm^−1^ (DXR Raman microscope, Thermo Scientific) with a resolution of 4 cm^−1^. The 532 nm line of a diode-pumped solid-state high-brightness laser was used as the excitation source. Conventional and high-resolution transmission electron microscopy (TEM/HRTEM) analyses were performed on an FEI Talos F200X microscope (Thermo Fisher Scientific, Waltham, MA, United States) at 200 kV. Samples for TEM analysis were prepared by dispersing the powders in ethanol by ultrasound. After dispersion, a drop of the solution was placed on a carbon-coated copper grid and dried in air. The recorded TEM micrographs were used to estimate the mean particle size by measuring 100–200 particles using ImageJ software (NIH, Bethesda, MD, United States). The particle morphologies were characterized by field emission scanning electron microscopy (FE-SEM, Ultra plus, Carl Zeiss, Germany). Samples for FE-SEM analysis were dispersed in water in an ultrasonic bath for 30 min; after dispersion, a few drops were filtered through a cellulose acetate membrane. The membrane was placed on carbon tape on the aluminum stub and coated with carbon for electron reflection. Before analysis, the samples were vacuumed for 15 min. Energy-dispersive X-ray fluorescence (EDX) spectroscopy with elemental maps was performed on an FEI SCIOS 2 Dual Beam electron microscope (Thermo Fisher Scientific, Waltham, MA, United States) operated at 10 kV. UV-Vis DRS were recorded using an Agilent Cary 5000 spectrophotometer equipped with a diffuse reflectance accessory. The measurements were performed in the range of 800–200 nm with a data interval of 1 nm and a scan rate of 600 nm/min, using a commercial PTFE standard for baseline correction. The PL spectra were recorded on a Horiba Jobin Yvon Fluorolog FL3–22 spectrofluorometer using Xe lamp excitation.

### 2.4 Photo-electrochemical activities of the catalysts

The electrocatalytic (EC) activities of the prepared samples were tested using linear sweep voltammetry (LSV). The EC measurements were performed on an Ivium VertexOne potentiostat/galvanostat in a conventional three-electrode quartz cell consisting of a glassy carbon as a working electrode, a platinum foil as a counter electrode, and a saturated calomel electrode (SCE) as a reference electrode. The tests were carried out in both acidic and alkaline electrolytes. An aqueous solution of 0.1 M H_2_SO_4_ (p.a. Merck), pH ∼ 1, was used as the acidic electrolyte, while 0.1 M NaOH (p.a. Merck), pH ∼ 13, was used as the alkaline electrolyte. The working electrode with a surface area of about 0.3 cm^2^ was coated with catalyst ink prepared by mixing 5 mg of a catalyst powder with 10 μL of 5% Nafion solution (Ion Power, United States) as a binder, 50 μL ethanol, and 50 μL water. This slurry was homogenized in an ultrasonic bath for 45 min. Subsequently, 5 μL of the prepared ink was coated as a thin film on the working electrode. To evaporate the solvent, the electrode was dried for 45 s with an infrared lamp (Tungsram INFRASEC 250 W). Measurements were performed in a potential range from 0.2 to 1.9 V vs. SCE for the OER reaction and from 0.2 to −1.9 V vs. SCE for the HER reaction with a scan rate of 20 mV⋅s^−1^. The measurements were performed in the dark and under illumination, after 60 min of exposure to an Osram Ultra-Vitalux 300 W lamp. The distance between the cell and the light source was set to 15 cm, which resulted in a light intensity of approximately 100 mW⋅cm^−2^.

All potentials were measured against the SCE and converted to a reversible hydrogen electrode (RHE) scale using Eq. 1:

$$E_{RHE}\;\left(V\right)=E_{SCE}+0.244+0.059\;pH.$$ (1)

To ensure gas saturation for the ORR tests, high-purity O_2_ gas (99.998%) was bubbled into the electrolyte solution for 15 min before and during the electrochemical measurements. The LSV was measured in the potential range between 0.2 and 1.2 V vs. RHE with a scan rate of 5 mV⋅s^–1^. The overpotential (η) was defined as μ = E (vs. RHE)–1.23 V. The number of electrons transferred in the ORR process was calculated using the Koutecky–Levich equation (Eq. 2):

$$\frac{1}{j}=\frac{1}{j_{k}}+\frac{1}{B\omega^{1/2}}.$$ (2)

Parameter B is defined as Eq. 3:

$$B=0.62nFv^{-1/6}C_{O_{2}}{D_{O_{2}}}^{2/3},$$ (3)

where *n* is the number of electrons exchanged per molecule of O_2_, *F* is the Faraday constant, *υ* is the kinematic viscosity of the electrolyte 0.01 cm^2^⋅s^−1^, *C*_O2_ is the bulk concentration of O_2_ 1.2⋅10^−3^ mol⋅dm^−3^, and *D*_O2_ 1.9⋅10^−5^ cm^2^⋅s^−1^ is the diffusion coefficient of O_2_ in 0.1 M NaOH (Mladenović et al., 2020). The sample suspension (10 μL) was loaded on the glassy carbon (GC) rotating disc electrode (RDE), which was used as the working electrode (surface 0.19625 cm^2^). The LSV measurements were performed in an O_2_-saturated 0.1 M NaOH aqueous solution at different rotation speeds (from 200 to 1,600 rpm) at a scan rate of 10 mV s^−1^ from 0.2 to 1.2 V vs. RHE.

The electrochemical reaction kinetics were analyzed using Tafel plots based on the Tafel equation (Eq. 4):

$$\eta =b\cdot \log\left(j/j_{o}\right),$$ (4)

where *η* is the overpotential, *b* is the Tafel slope (mV⋅dec^−1^), *j* is the current density, and *j*_o_ is the exchange current density (mA⋅cm^−2^).

The electrochemical active surface area (ECSA) was determined by the electrochemical double-layered capacitance (*C*_*dl*_) using Eq. 5:

$$ECSA=C_{dl}/C_{s},$$ (5)

where *C*_*s*_ is the specific capacitance of the catalyst; usually 0.035 mFcm^–2^ and 0.040 mFcm^–2^ in 1 M H_2_SO_4_ and 1 M NaOH, respectively (McCrory et al., 2013). The *C*_dl_ was calculated through cyclic voltammetry (CV) for all electrocatalysts in both electrolytes. CVs were performed in a non-Faradaic region at seven different scan rates (20, 40, 60, 80, 100, 150, and 200 mV s^-1^). The average current densities (*Δj* = (*j*_a_−*j*_c_)/2) were plotted as a function of the scan rate, and *C*_*dl*_ was determined as the slope of the linear fit (Perez Bakovic et al., 2021).

## 3 Results and discussion

### 3.1 Catalyst characterization

**FIGURE 1:**
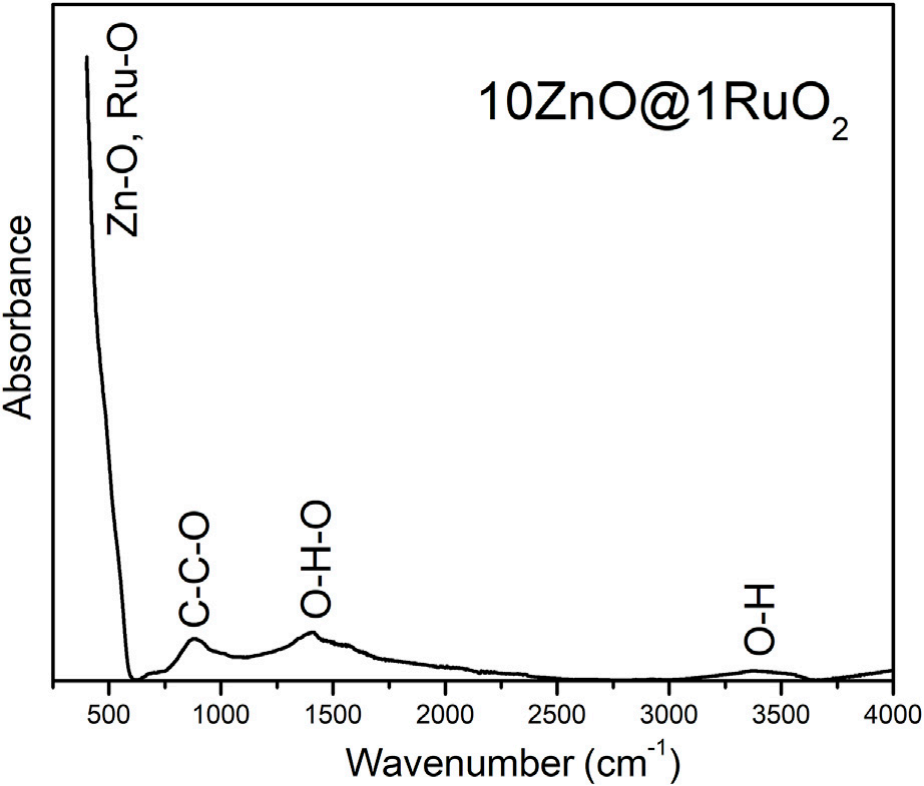
FTIR spectrum of pristine 10ZnO@1RuO_2_ particles.

ATR/FTIR spectroscopy analysis of the surface functional groups of the as-prepared 10ZnO@1RuO_2_ particles was used to identify the possible residues of the reagents used for MW processing. As shown in Figure 1, the most prominent band was in the range 400–600 cm^–1^ and was attributed to the superposition of Zn–O vibrations in the ZnO lattice (Sadollahkhani et al., 2014; Marković et al., 2019) and Ru–O vibrations in the RuO_2_ lattice (Khorshidi and Sadeghi, 2016). The weak peak at 890 cm^–1^ was attributed to the symmetric stretching vibrations of –C–C–O, most probably due to the residue of the ethanol used to rinse the sample after microwave processing (Smith, 1998; Chen et al., 2017). The broad bands in the 1,320–1,575 and 3,200–3,550 cm^–1^ ranges were attributed to the bending and stretching vibrations, respectively, of the O–H groups of the adsorbed water molecules, which were hydrogen bonded to the surface of the composite particles (Marković et al., 2019). Thus, the FTIR spectrum indicated that a negligible amount of hydroxyl groups was adsorbed on the surface of the as-prepared composite particles without residues of the processing reagents.

**FIGURE 2:**
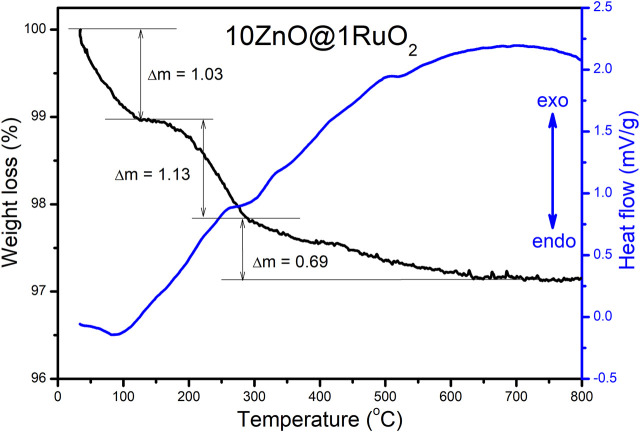
TG-DTA plots of 10ZnO@1RuO_2_ particles.

The thermal behavior of the 10ZnO@1RuO_2_ composite was investigated by TG-DTA. The TG curve presented in Figure 2 indicates an overall weight loss of 2.85%; the weight loss of about 1% in the temperature range from 30°C to 125°C was probably due to a small amount of surface water and ethanol remaining after the synthesis procedure while the further drop of 1% in the temperature range from 125°C to 260°C was attributed to adsorbed gasses. The temperature range from 260°C to 800°C showed a continuous weight loss of approximately accompanied by a broad exothermic peak in the DTA curve; this weight loss was probably due to the ordering of the crystal structure.

The annealing of metal oxide composites can significantly affect their (photo)electrocatalytic activity, in which the annealing temperature has a large impact (Marković et al., 2017). To determine the impact of oxygen vacancies on the (photo)electrocatalytic activity of the ZnO@RuO_2_ composite, we annealed the as-prepared composite at two different temperatures. Based on the TG-DTA results, the temperatures chosen to anneal the as-prepared composite were 300°C, since adsorbed moisture and gasses will be desorbed up to this temperature, and 600°C as the temperature that allows ordering of the crystal structure without a phase transformation or the formation of new phases.

**FIGURE 3:**
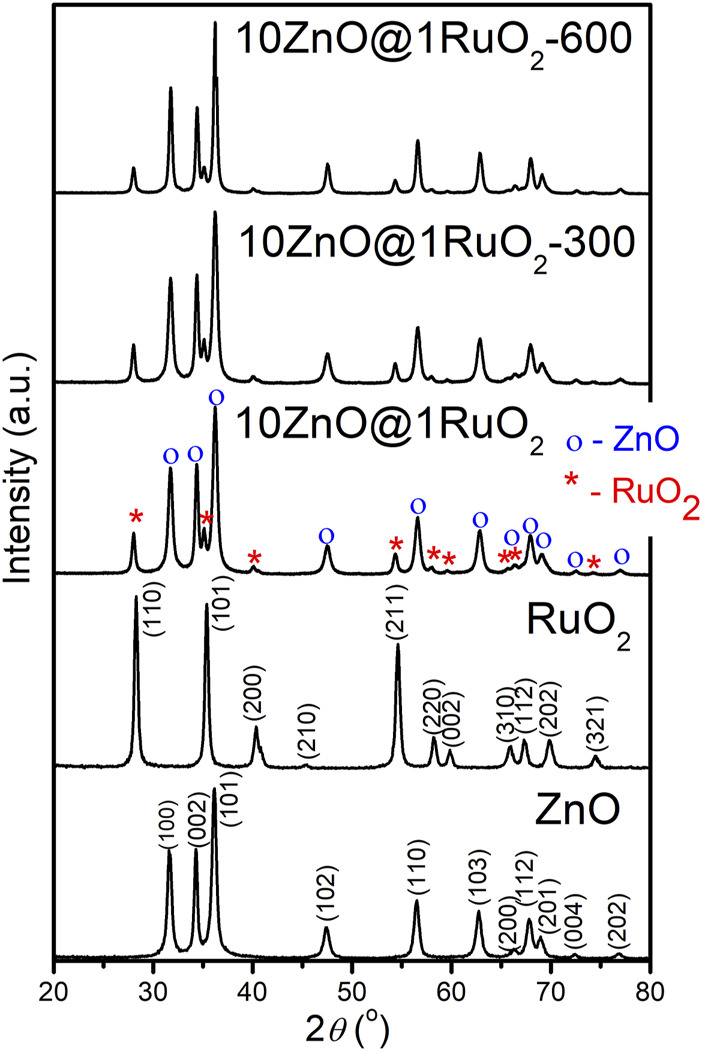
XRD patterns of the catalyst particles.

The phase composition of the catalysts and the evolution of the crystal structure as a function of annealing temperature were studied by X-ray diffraction (XRD). For comparison, besides the XRD patterns of the ZnO@RuO_2_ composites, Figure 3 also shows the XRD patterns of ZnO, synthesized by the same procedure as the composites, and bare RuO_2_. The XRD pattern of the pristine ZnO indicates a pure, highly crystalline, wurtzite-type structure with a hexagonal phase (*P*6_3_ *mc* space group; COD no. 96-230-0113), while the diffraction peaks in the XRD pattern of bare RuO_2_ are a rutile-type structure with a tetragonal phase (*P*4_2_/*mnm* space group; COD no. 96-900-7542). The ZnO@RuO_2_ composite powder consisted of hexagonal ZnO and tetragonal RuO_2_ without other crystal phases. According to the XRD patterns, the composites annealed at 300°C and 600°C had the same phase compositions as the as-prepared ZnO@RuO_2_ composite, but with slightly increased crystallinity from 92.15% for ZnO@RuO_2_-300% to 94.5% for ZnO@RuO_2_-600, which correlated with the weight loss of about 0.6% accompanying the large exothermic peak from 300°C to 600°C in the TG-DTA graphs (Figure 2).

The calculated unit cell parameters, average crystallite size, and degree of crystallinity of pristine ZnO, bare RuO_2_, and the prepared composites are listed in Table 1. The calculated unit cell parameters for pristine ZnO and bare RuO_2_ powders agree well with the values reported in the corresponding COD cards (96-230-0113 and 96-900-7542, respectively). The calculated values showed that additional annealing caused a reduction of the ZnO and RuO_2_ crystal lattices in the ZnO@RuO_2_ composite. Moreover, the results indicated an increase in the average crystallite size of the zinc oxide after 1 h of annealing at 300°C and promoted growth at 600°C, while the average crystallite size of RuO_2_ was slightly reduced during annealing at 600°C. In addition, the crystallinity increased with increasing annealing temperature.

**TABLE 1:** Crystallinity values, average crystallite size, unit cell parameters with standard deviations, and mean particle size determined from the TEM images.

Sample	Crystallinity (%)	Average crystallite size (nm)		Unit cell parameters (Å)		*d*m^a^ (nm)
		ZnO	RuO_2_	*a* = *b*	*C*	
ZnO	90.3	17.1	—	3.2515 (8)	5.2110 (2)	21.9 ± 4.9
RuO_2_	92.1	—	18.3	4.4975 (9)	3.1017 (1)	27.6 ± 6.2
ZnO@RuO_2_	88.8	16.8	21.5	ZnO		26.6 ± 7.3
				3.2515 (8)	5.2108 (3)	
				RuO_2_		
				4.4961 (1)	3.1010 (3)	
ZnO@RuO_2_-300	92.2	17.8	21	ZnO		25.9 ± 6.3
				3.2495 (1)	5.2076 (4)	
				RuO_2_		
				4.4945 (1)	3.1003 (3)	
ZnO@RuO_2_-600	94.5	22	20	ZnO		43.4 ± 13.4
				3.2484 (7)	5.20419 (2)	
				RuO_2_		
				4.4931 (1)	3.1006 (2)	

^a^ *d*m, mean particle size determined from TEM images.

Interfacial substitution of zinc sites in ZnO with a crystal radius of Zn^2+^ in the coordination IV equal to 0.74 Å (Shannon, 1976) by Ru^4+^ with a crystal radius in the coordination IV equal to 0.76 Å (Shannon, 1976) that diffused from RuO_2_ is expected to occur during the annealing process. However, due to the small amount of RuO_2_ compared to ZnO and almost the same crystal radii, the change in interplanar spacing cannot be detected by XRD.

**FIGURE 4:**
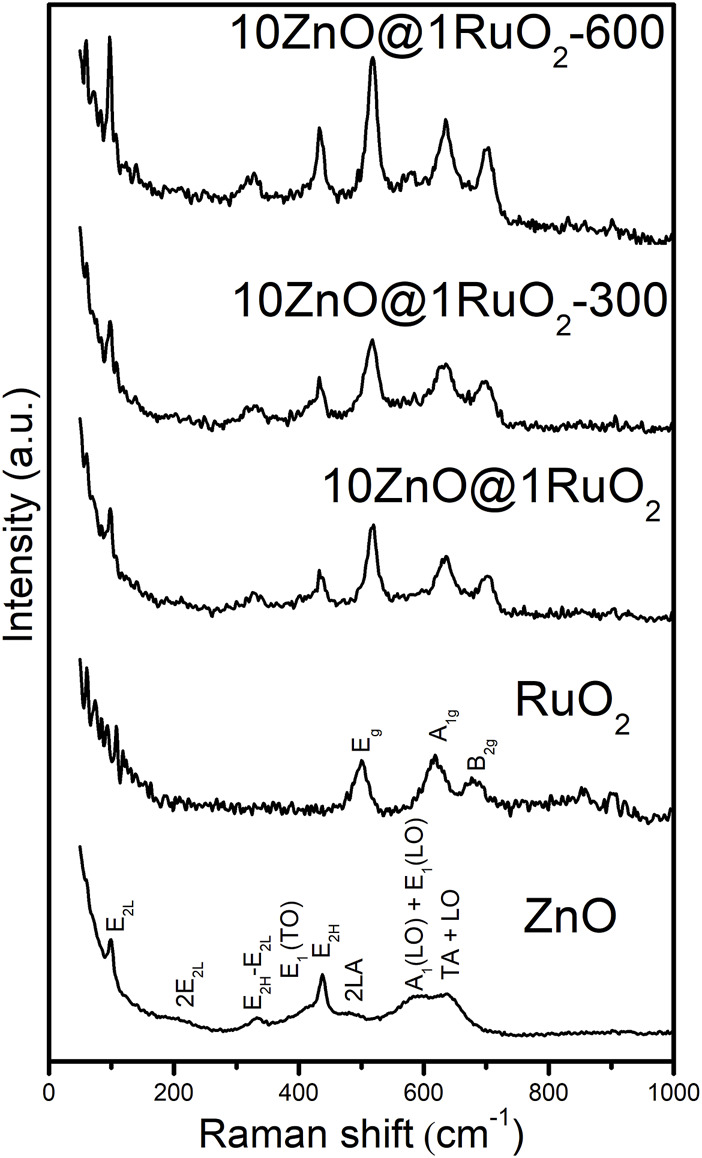
Raman spectra of the catalyst particles.

Raman spectroscopy was employed to reveal the annealing-induced changes in local order and defect state in the catalyst’s crystal lattice (Figure 4). The two most intense bands in the Raman spectrum of pristine ZnO were associated with the E_2_ modes; the first band at 98 cm^–1^, attributed to the non-polar optical mode E_2L_, occurred due to the vibration of the heavy Zn sub-lattice; the second, sharp peak, centered at 436 cm^–1^ was assigned to the E_2H_ optical mode and occurred due to oxygen atom vibration (Sanchez Zeferino et al., 2011; Ghosh et al., 2018). A weak shoulder at 218 cm^–1^ and a low-intensity peak at 330 cm^–1^ were attributed to the second-order phonon mode 2E_2L_, and the zone boundary multiphonons E_2H_–E_2L_, respectively (Sanchez Zeferino et al., 2011). The shoulder at 405 cm^–1^, assigned to the E_1_ (TO) mode, indicated that the ZnO crystallites did not exhibit elongation in the *c*-axis direction typical of ZnO particles (Li et al., 2010). A broad band in the spectral range 510–720 cm^–1^ represented a combination of two bands; the first, centered near 570 cm^–1^, occurred due to a longitudinal optical (LO) mode consisting of A_1_ (at 574 cm^–1^) and E_1_ (at 587 cm^–1^) modes, while the other at 635 cm^–1^ occurred due to a combination of acoustic and optical modes (TA + LO). The A_1_ (LO) mode was accompanied by a broad band of low intensity near 480 cm^–1^, denoted as 2LA, which was ascribed to the interfacial surface phonon mode, characteristic of surface defects (Cuscó et al., 2007; Sanchez Zeferino et al., 2011). Two LO modes may be ascribed to bulk defects such as oxygen vacancies. The presence of impurities and defects strongly influences both LO modes, especially E_1_ (LO). The relatively high-intensity A1 (LO) + E1 (LO) modes in the Raman spectrum of pristine ZnO (Figure 4) suggest a certain amount of intrinsic bulk defects caused by rapid crystallization driven by microwave irradiation (Marković et al., 2019; Rajić et al., 2020). The phonon modes observed in the Raman spectrum of pure ZnO were typical of wurtzite ZnO with a space group of $C_{6v}^{4}$ (Pimentel et al., 2014). Three major features in the Raman spectrum of bare RuO_2_, the, E_g,_ A_1g_, and B_2g_ modes, are located at 528, 646, and 716 cm^–1^, respectively (Chen et al., 2004). All the peaks in the Raman spectra of the 10ZnO@1RuO_2_ composites could be attributed to ZnO and RuO_2_, with no peaks related to impurities. The disappearance of the 2LA Raman mode with composite annealing suggested the disappearance of surface defects, while the more intense and narrower E_2H_ band indicates increased crystallinity with more ordered local symmetry. A slight blue shift in the Raman peak positions and line narrowing of the RuO_2_ particles in the annealed composites relative to those of the as-prepared 10ZnO@1RuO_2_ indicate increased crystallinity and particle size.

**FIGURE 5:**
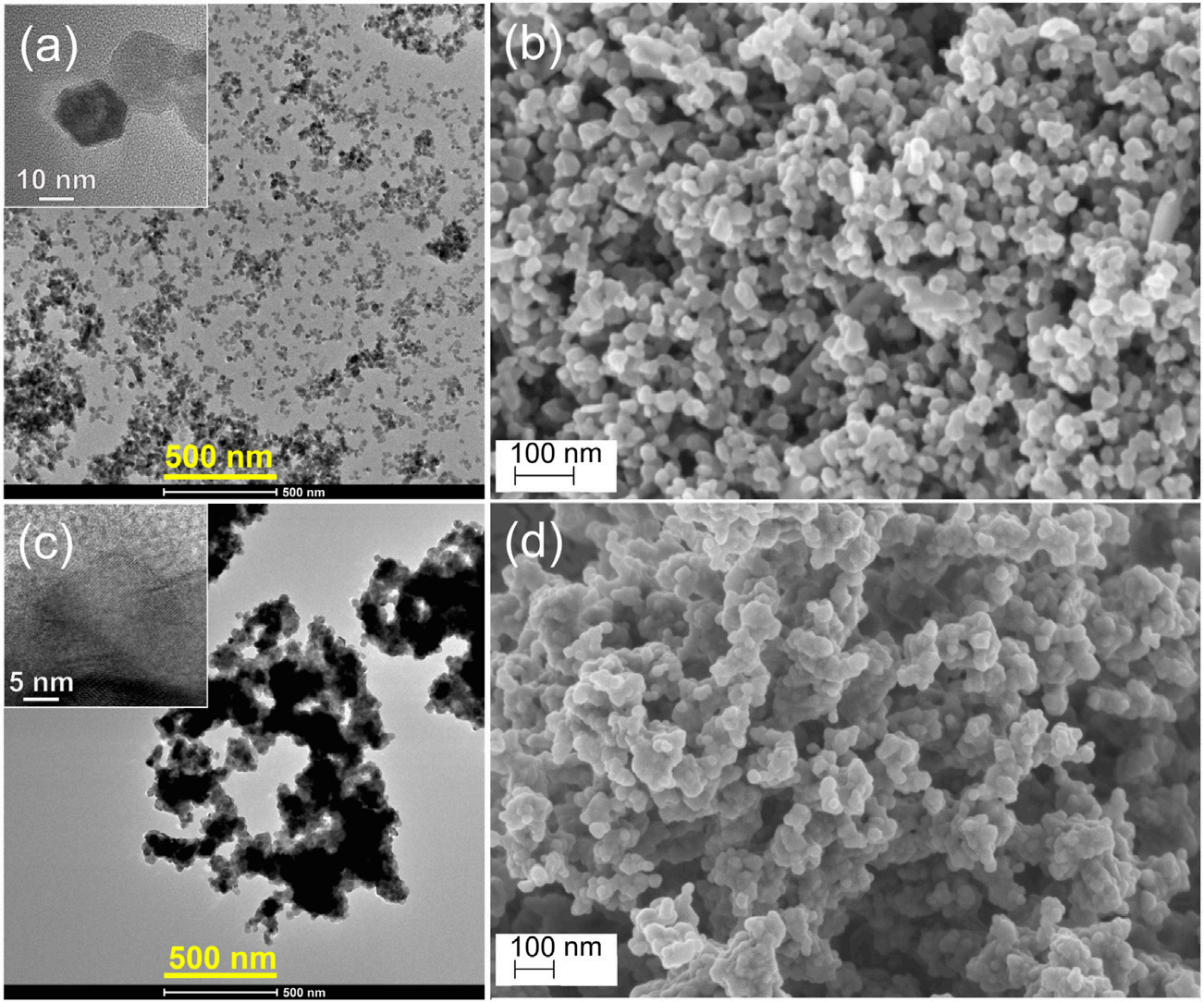
TEM/HRTEM and FESEM images of **(A, B)** pristine ZnO, and **(C, D)** bare RuO_2_ particles. Bar is 500 nm in the TEM images and 100 nm in the FESEM images.

**FIGURE 6:**
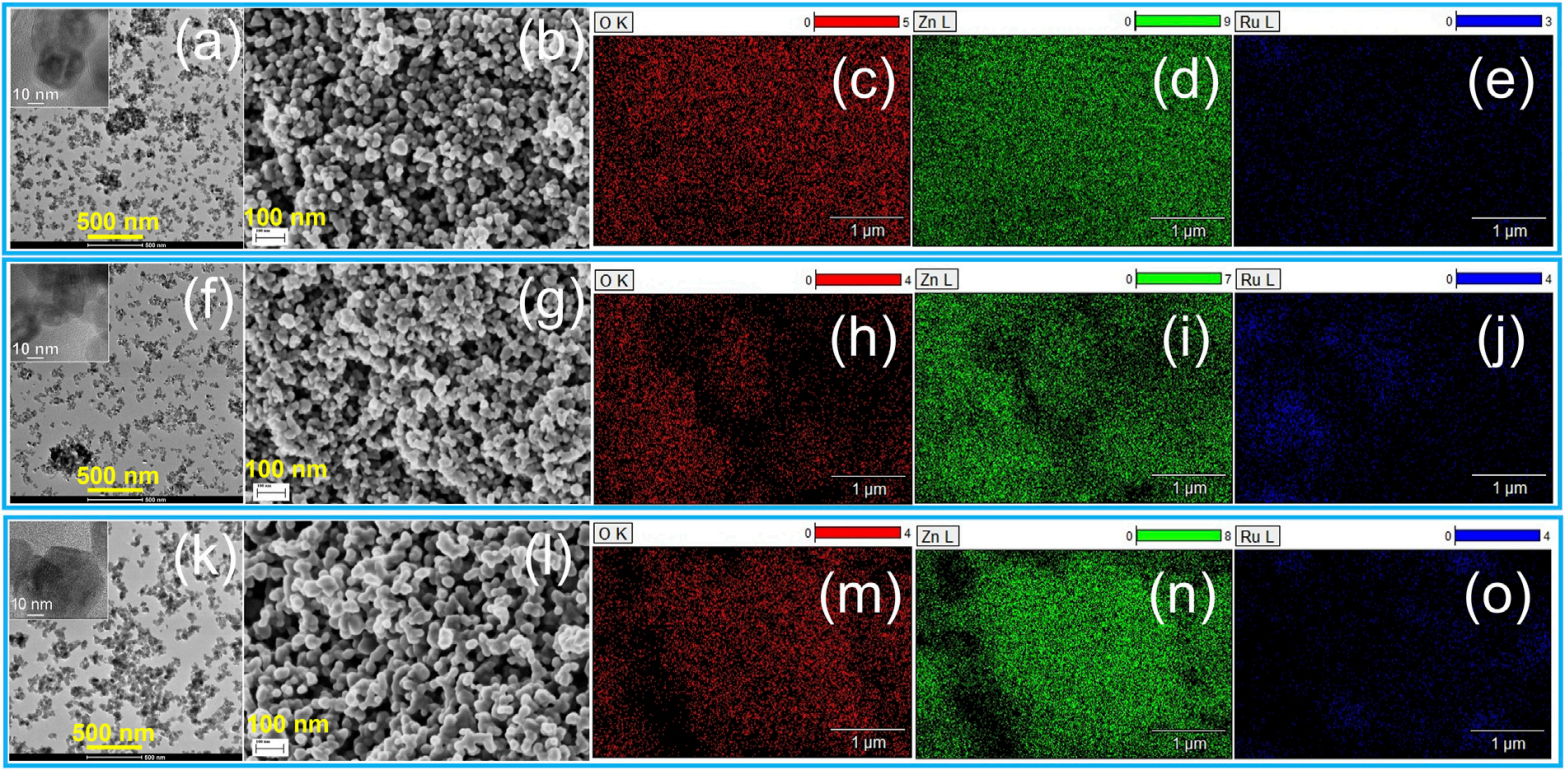
TEM/HRTEM and FESEM images with compositional EDX maps of Zn, Ru and O, of the 10ZnO@1RuO_2_ particles: **(A–E)** as pristine, **(F–J)** annealed at 300, and **(K–O)** annealed at 600°C. Bar is 500 nm in the TEM images and 100 nm in the FESEM images.

**FIGURE 7:**
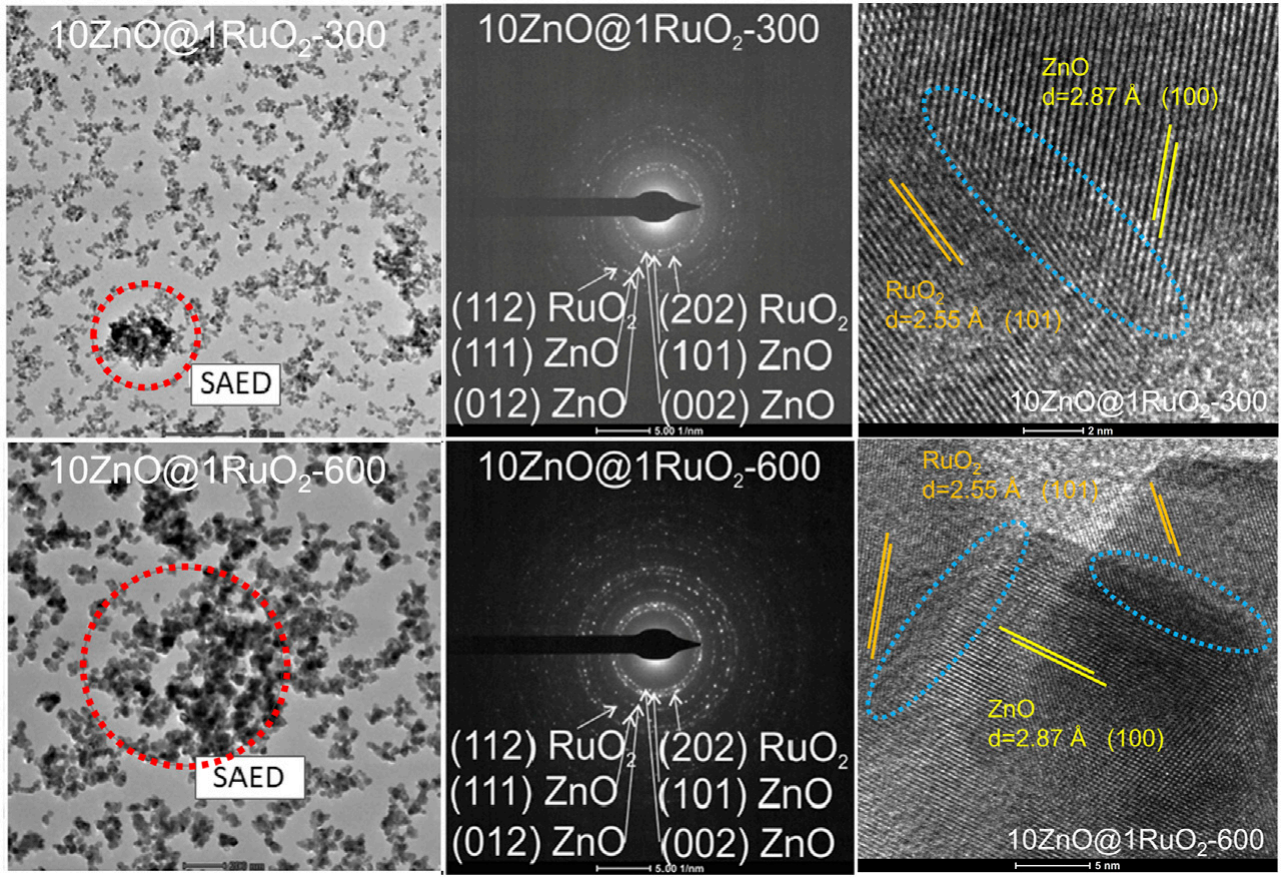
TEM, corresponding SAED pattern, and HRTEM with marked heterojunctions of 10ZnO@1RuO_2_-300 and 10ZnO@1RuO_2_-600 powders.

To gain further insight into the crystal structure and morphology of the ZnO, RuO_2_, and 10ZnO@1RuO_2_ particles, TEM/HRTEM and FESEM analyses were performed. The TEM micrographs with the HRTEM images as inset and FESEM micrographs of ZnO and RuO_2_ particles are presented in Figures 5A–D. The HRTEM images (Supplementary Figure S1) confirm the good crystallinity; the observed lattice planes with a *d* spacing of 2.87 Å correspond to the (100) planes of ZnO, while the *d* spacing of 2.55 Å corresponds to the (101) planes of RuO_2_. The TEM analysis showed that the ZnO particles (Figure 5A) were less agglomerated than the RuO_2_ particles (Figure 5C). The FESEM micrographs suggested that the ZnO particles were more softly agglomerated compared to RuO_2_ particles (Figures 5A, B, respectively). Figure 6 displays the TEM/HRTEM and FESEM micrographs of the 10ZnO@1RuO_2_ composite particles, as-prepared and annealed at 300°C and 600°C. The TEM images (Figures 6A, F, K) show that when ZnO and RuO_2_ are combined in the 10ZnO@1RuO_2_ composite, the agglomeration is more similar to ZnO than to RuO_2_ (Figure 5A, C). The TEM and SAED images (Supplementary Figure S1 and Figure 7) suggested good crystallinity of the prepared composites. The presence of the diffraction ring associated with (002), (101), (012), and (111) crystallographic planes of the ZnO phase and (202) and (112) crystallographic planes of the RuO_2_ phase was confirmed in 10ZnO@1RuO_2_-300 and 10ZnO@1RuO_2_-600 samples. Heterojunctions formed during annealing at the interface of two grains of different phases were evidenced in HRTEM images (Figure 7). The TEM images were used to estimate the mean particle size; the corresponding particle size distribution histograms are presented in Supplementary Figure S2 (in Supplementary Material) while the mean particle size values calculated from the histograms are listed in Table 1. These results showed that annealing at 300°C did not significantly affect the mean particle size, while increasing the annealing temperature to 600°C led to an increased mean particle size from 25.9 ± 6.3 to 43 ± 13.4. The FESEM micrographs (Figures 6B, G, L) showed that the morphology of the catalysts was not significantly affected by the annealing processes. These micrographs indicated hardening of the agglomerates with annealing compared to the soft agglomerates observed in the as-prepared composite. The distributions of Zn, Ru, and O ions in 10ZnO@1RuO_2_ composite particles were investigated by energy-dispersive X-ray spectroscopy mappings in SEM, Figure 6. The mappings of Zn, Ru, and O as individual elements confirmed the uniform distribution of the ions in all three composite samples.

**FIGURE 8:**
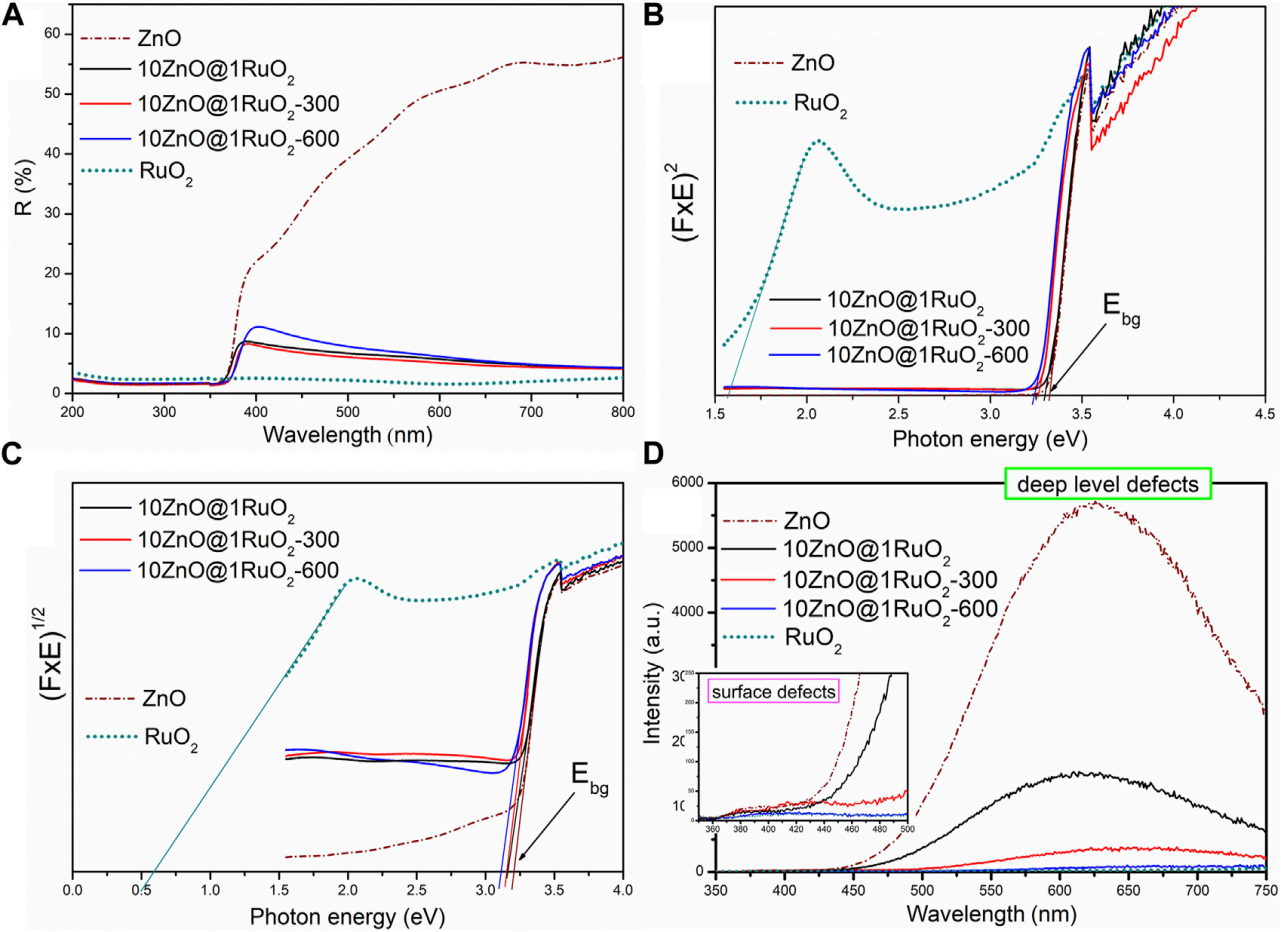
**(A)** UV-Vis DR spectra, **(B)** Kubelka–Munk plots for direct, **(C)** indirect bandgap, and **(D)** PL spectra of the catalyst particles.

The optical properties were studied by UV-Vis diffuse reflectance (DRS) and photoluminescence (PL) spectroscopy (Figure 8). Figure 8A shows the UV-Vis DRS spectra of the examined catalysts. The ZnO-based catalysts showed reflectance spectra with band edge absorption near 380 nm, which is quite different from the spectra of bare RuO_2_, whose reflectance is almost negligible in the whole spectral range studied. In the visible range, ZnO reflected 20%–55% of light, while the as-prepared 10ZnO@1RuO_2_ composite reflected <10% of visible light. For example, at 550 nm, the reflectance dropped from 45.6% for ZnO via 6.3% for the as-prepared 10ZnO@1RuO_2_ composite to 1.7% for RuO_2_. Accordingly, the absorption capacity of the 10ZnO@1RuO_2_ composite was significantly improved compared to pristine ZnO. The improved absorption capacity of the composite may be attributed to a small amount of RuO_2_ which absorbed about 98% of light in the examined spectral range. (In this study, the absorption capacity can be correlated with reflectance since the UV-Vis DRS measurements were determined on 1 mm-thick pellets; thus, transparency could be ignored). Furthermore, additional annealing of the composite had a negligible effect on the percentage of reflectance in the spectral range of 380–700 nm. The Kubelka–Munk function was applied to determine the direct and indirect band gap energies (*E*_bg_) of the catalyst particles (Marković et al., 2019; Rajić et al., 2020). These energies were estimated by extrapolating the linear part of the curves [*F*(R) × *E*]^2^, i.e., [*F*(R) × *E*]^1/2^ with respect to *E* (eV) to 0, Figures 8B, C, respectively. The estimated values of the direct band gap energies of ZnO, RuO_2_, 10ZnO@1RuO_2_, 10ZnO@1RuO_2_-300, and 10ZnO@1RuO_2_-600 were 3.32, 1.57, 3.30, 3.26, and 3.24 eV, respectively. The estimated values of the indirect band gap energies of ZnO, RuO_2_, 10ZnO@1RuO_2_, 10ZnO@1RuO_2_-300, and 10ZnO@1RuO_2_-600 were 3.20, 0.60, 3.16, 3.15, and 3.11 eV, respectively.

Room-temperature PL spectroscopy was used to study the effects of annealing temperature on point defects in the 10ZnO@1RuO_2_ crystal lattice. The PL emission spectra of all the catalysts studied (Figure 8D) showed a broad emission band centered near 630 nm, which can be attributed to bulk defects, more specifically to bulk oxygen vacancies (Ahn et al., 2009; Marković et al., 2019; Rajić et al., 2020). The low intensity of the violet-blue emission band at 420 nm (inset in Figure 8D) indicated negligible surface defects compared to the bulk defects (Marković et al., 2019; Rajić et al., 2020). The most intense PL spectrum was observed for pristine ZnO nanoparticles, while the spectrum with the lowest intensity was observed for bare RuO_2_. As we previously reported, the large number of bulk defects in ZnO occurs due to the rapid crystallization caused by the high energy delivered to the Zn(OH)_2_ precipitate via microwave irradiation (Marković et al., 2019; Rajić et al., 2020). In contrast, a negligible number of bulk defects characterizes commercially available RuO_2_. As shown in Figure 8D, the as-prepared 10ZnO@1RuO_2_ composite exhibited an almost five-fold lower PL intensity than ZnO, indicating that ZnO particles precipitated and microwave processed in the presence of a small amount of RuO_2_ (ZnO:RuO_2_ = 10:1) had a significantly lower number of bulk defects in the crystal structure. Moreover, additional annealing of the as-prepared 10ZnO@1RuO_2_ composite significantly affected the bulk defects, indicating that increased annealing temperature reduced the number of bulk oxygen vacancies to a greater extent.

### 3.2 Photo-electrochemical activity of the catalysts

The synthesized catalysts were used to prepare (photo)electrodes, which were analyzed as both (photo)anode and (photo)cathode depending on the applied potential interval. The overall water-splitting reaction can be expressed as two half-reactions, the mechanism of which depends on the reaction conditions, i.e., on the nature of the electrolyte (acidic or alkaline) (Wan et al., 2022):

In an acidic electrolyte:

$$\begin{array}{c} \left(HER\;on\right)\;Cathode:4H^{+}+4e^{-}\;\to \;2H_{2}\\ \left(OER\;on\right)\;Anode:2H_{2}O\;\to \;O_{2}+4H^{+}+4e^{-}\\ Total:2H_{2}O\;\to \;2O_{2}+2H_{2} \end{array}$$

In an alkaline electrolyte:

$$\begin{array}{c} \left(HER\;on\right)\;Cathode:4H_{2}O+4e^{-}\;\to \;2H_{2}+4{OH}^{-}\\ \left(OER\;on\right)\;Anode:4{OH}^{-}\;\to \;O_{2}+2H_{2}O+4e^{-}\\ Total:2H_{2}O\;\to \;2O_{2}+2H_{2} \end{array}$$

**FIGURE 9:**
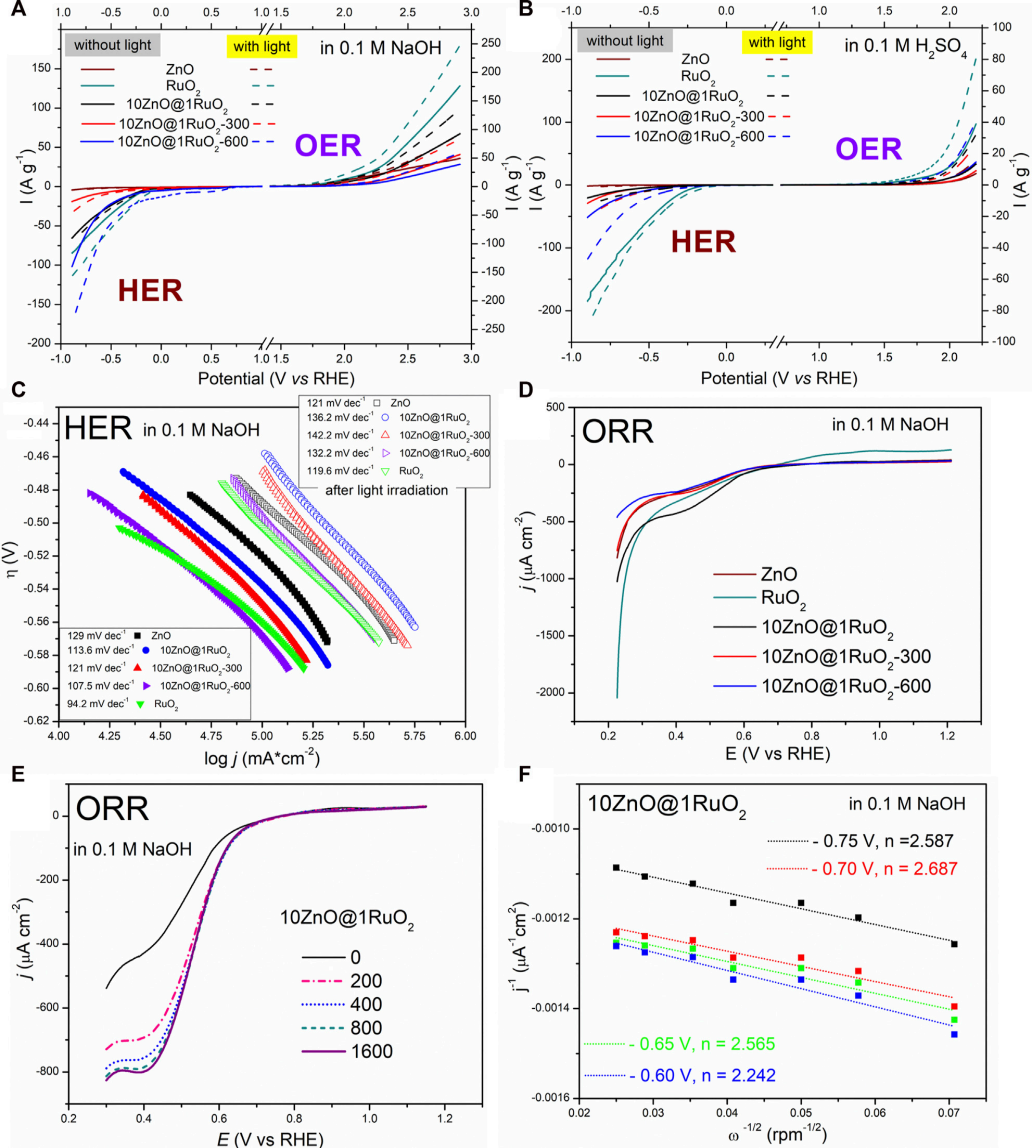
HER/OER LSVs of the catalyst photoelectrodes in **(A)** 0.1 M NaOH and **(B)** 0.1 M H_2_SO_4_ solution, at a scan rate of 20 mV s^−1^ in the dark condition and under light illumination. **(C)** Tafel plots derived from the LSVs in **(A)**. **(D)** ORR LSVs for the catalysts in 0.1 M NaOH. **(E)** ORR LSVs for 10ZnO@1RuO_2_ in 0.1 M NaOH at rotation rates of 200–1,600 rpm and a scan rate of 5 mV⋅s^−1^. **(F)** Koutecky–Levich plot of 10ZnO@1RuO_2_ based on the ORR polarization curves in **(E)**.

Two half-reactions, HER and OER, are commonly studied in alkaline and acidic media (Danilovic et al., 2013; Wang et al., 2021b). Many published data exist for ZnO-based catalysts in alkaline media (Yu et al., 2018) and few in acidic media (Uma et al., 2020; Pataniya et al., 2021). Therefore, we investigated the catalytic activity of the prepared composites toward HER and OER in both media. Linear sweep voltammetry (LSV) was employed to investigate the electrocatalytic activity of the prepared composites toward HER and OER. The HER and OER activities of the composites were evaluated according to onset potential and current density values. To estimate the photo-electrocatalytic activities of the composites, LSV measurements were performed in the dark and under illumination after 60 min of exposure. The results obtained for the composites were compared with those for pristine ZnO and bare RuO_2_. Figure 9A shows the LSV curves for the catalysts in 0.1 M NaOH electrolyte solution at a scan rate of 20 mV s^−1^. The estimated onset potentials at 10 A g^−1^ for OER in alkaline solution were 2.08, 2.06, 2.25, 2.36, and 1.94, for ZnO, 10ZnO@1RuO_2_, 10ZnO@1RuO_2_-300, 10ZnO@1RuO_2_-600, and RuO_2_, respectively. The same activity was observed when the current density values were compared at 2.9 V vs. RHE: 36.1, 92, 55.6, 38.7, and 175.7 A g^−1^ for ZnO, 10ZnO@1RuO_2_, 10ZnO@1RuO_2_-300, 10ZnO@1RuO_2_-600, and RuO_2_, respectively. The same trend of catalytic activity was also observed for HER in the 0.1 M NaOH solution. ZnO showed low electrocatalytic activity (onset potential, −0.884 V vs. RHE at 7 A g^−1^) while the onset potentials at −10 A g^−1^ of 10ZnO@1RuO_2_, 10ZnO@1RuO_2_-300, 10ZnO@1RuO_2_-600, and RuO_2_ were −0.289, −0.671, −0.284, and −0.251 V vs. RHE, respectively. The current densities at 0.9 V vs. RHE were 5.9, 65.1, 18.9, 103.2, and 84.7 mA cm^-2^ for ZnO, 10ZnO@1RuO_2_, 10ZnO@1RuO_2_-300, 10ZnO@1RuO_2_-600, and RuO_2_, respectively.

After 60 min of light irradiation, during the analysis of HER and OER in 0.1 M NaOH, the onset potentials for all samples shifted to lower values and the current densities increased. This indicated that photocarriers were formed in the catalysts after light exposure, which enhanced the reduction process of the adsorbed proton (Biroju et al., 2017). The most significant effect for HER was observed in the 10ZnO@1RuO_2_-600 catalysts, where the onset potential was reduced by 0.163 V. For OER, the onset potential shifted by a similar value in the 10ZnO@1RuO_2_-300 sample. The same trend was observed for the photocurrent. The 10ZnO@1RuO_2_-600 sample showed a current of 175.9 Ag^−1^ in the case of HER and 134.9 Ag^−1^ for OER at a potential of 2.9 vs. RHE.

Regarding the OER, in addition to the number of active sites on the electrocatalyst surface, the oxidation state of these active sites also plays an important role, with high oxidation states favoring OER (Milikić et al., 2023). In the acidic electrolyte solution, the OER activities of the studied catalysts were similar (Figure 9B). The estimated onset potentials for OER in the 0.1 M H_2_SO_4_ solution were 2.15, 2.21, 2.14, and 1.97 V vs. RHE for 10ZnO@1RuO_2_, 10ZnO@1RuO_2_-300, 10ZnO@1RuO_2_-600, and RuO_2_, respectively. For ZnO, the onset potential was 2.196 V vs. RHE at 6.7 A g^−1^. Comparisons of current density values at 2.2 V vs. RHE showed similar activity in the dark for all composite samples (13 Ag^−1^). Upon light irradiation, the onset potential of the annealed composites, 10ZnO@1RuO_2_-300 and 10ZnO@1RuO_2_-600, shifted to lower values (0.180 V), while the onset potential of the as-prepared 10ZnO@1RuO_2_ composite decreased to approximately 0.140 V. The highest photocurrent of 41.1 Ag^−1^ was observed for 10ZnO@1RuO_2_-600. Regarding HER in the 0.1 M H_2_SO_4_ solution, the onset potential at −10 A g^-1^ were −0.714, −0.689, −0.537, and −0.279 V vs. RHE for ZnO, 10ZnO@1RuO_2_, 10ZnO@1RuO_2_-300, 10ZnO@1RuO_2_-600, and RuO_2_, respectively.

Regarding the catalytic activity toward HER, the lowest onset potential and the highest current are also registered for 10ZnO@1RuO_2_-600; the values obtained are −0.537 V vs. RHE and 51.2 Ag^−1^, respectively. All the samples show improved photocatalytic activity; in particular, both annealed composites had lower onset potentials of about 0.160 V, while the onset potential of the as-prepared composite shifted to a lower value of 0.188 V. The highest photocurrent of about 117.8 Ag^−1^ was observed for 10ZnO@1RuO_2_-600. The Tafel slope is an important parameter for evaluating the electrocatalytic kinetics of HER and OER reactions, in which a smaller Tafel slope indicates better electrocatalytic behavior for each of the reactions (Jin et al., 2018). Thus, to provide better insight into the kinetics and mechanism of the HER and OER catalytic reactions, we used Tafel plots (*η* vs. log*j*) (Figure 9C) based on the Tafel equation, (Eq. 4). The Tafel slopes were determined based on the linear segment in the low-potential region, in which the smallest *b* value indicated the best catalytic activity. From the Tafel slopes listed in Table 2, compared to all the reactions studied, the HER in the alkaline electrolyte had the fastest kinetics under both dark (∼110 mV⋅dec^−1^) and light (∼130 mV⋅dec^−1^) experimental conditions, while OER in the alkaline solution had the slowest reaction. The Tafel slopes also showed that the OER was much faster in the acidic electrolyte (∼110 mV⋅dec^−1^, in the dark, and ∼92–170 mV⋅dec^−1^ under light irradiation), while the HER was slightly slower in an acidic electrolyte compared to the HER in an alkaline medium, especially under light irradiation (∼250–380 mV⋅dec^−1^). The theoretical values of the Tafel slope for HER were 30, 40, and 120 mV dec^−1^ corresponding to Tafel, Heyrovsky, and Volmer reactions, respectively (Pataniya et al., 2021). In the present study, the Tafel slopes for HER in acid and alkaline media suggested the Volmer reaction mechanism both in the dark and during light irradiation. The kinetics of all the studied reactions, especially the OER, can be improved by using carbon materials as additives to the composites (Guan et al., 2018; Xie et al., 2020). The onset potential, current, and Tafel slope suggested that 10ZnO@1RuO_2_-600 was a good photo(electro) catalyst for HER in the alkaline and acidic mediums, as well as the OER in the acidic medium. The results of the comparisons of catalytic activities of different ZnO-based materials in terms of the onset potentials and Tafel slopes are listed in Table 2.

**TABLE 2:** Onset potentials (V vs. RHE) and Tafel slopes (mV⋅dec^−1^) of the tested catalysts toward HER and OER in alkaline and acidic media in the dark and after 60 min of light irradiation compared to literature data for different ZnO-based photocatalysts.

Catalyst	Electrolyte	Onset potential (V vs. RHE)		Tafel slope (mV⋅dec^−1^)		Reaction	Reference
		Dark	Light	Dark	Light		
ZnO	0.1 M NaOH	−0.884	−0.878	129	121	HER	This work
ZnO		2.08	2.03	640	549	OER	
10ZnO@1RuO_2_		−0.289	−0.126	113	136	HER	
10ZnO@1RuO_2_		2.06	1.96	255	507	OER	
10ZnO@1RuO_2_-300		−0.671	−0.562	121	142	HER	
10ZnO@1RuO_2_-300		2.25	2.09	268	495	OER	
10ZnO@1RuO_2_-600		−0.284	0.121	107	132	HER	
10ZnO@1RuO_2_-600		2.36	2.24	263	512	OER	
RuO_2_		−0.251	−0.152	94	119	HER	
RuO_2_		1.94	1.84	367	475	OER	
ZnO	0.1 M H_2_SO_4_	−0.777	−0.739	237	288	HER	
ZnO		2.196	2.11	170	189	OER	
10ZnO@1RuO_2_		−0.714	−0.526	152	380	HER	
10ZnO@1RuO_2_		2.15	2.01	113	179	OER	
10ZnO@1RuO_2_-300		−0.689	−0.526	134	274	HER	
10ZnO@1RuO_2_-300		2.21	2.03	111	94	OER	
10ZnO@1RuO_2_-600		−0.537	−0.377	114	250	HER	
10ZnO@1RuO_2_-600		2.14	1.96	92	92	OER	
RuO_2_		−0.279	−0.211	80	91	HER	
RuO_2_		1.97	1.84	87	81	OER	
ZnO	0.1 M KOH	−0.650	—	215	—	HER	Sun et al. (2021)
5% Ag–ZnO		−0.568	—	192	—	HER	Sun et al. (2021)
WS_2_/ZnO	0.5 M H_2_SO_4_	−0.201	−0.182	77	83	HER	Pataniya et al. (2021)
ZnO	0.5 M H_2_SO_4_	−0.210	—	—	—	HER	Uma et al. (2020)
ZnO/Fe_2_O_3_		−0.125	—	—	—	HER	Uma et al. (2020)
ZnO	0.1 M KOH	0.580	—	330	—	OER	Jang et al. (2015)
Mn-ZnO		0.460	—	70	—	OER	Jang et al. (2015)
IrO_2_−ZnO_12	0.5 M H_2_SO_4_	1.481	—	42.9	—	OER	Jin et al. (2022)
ZnO	0.1 M Na_2_SO_4_	2.001	—	287	—	OER	Rajić et al. (2020)
ZnO:10Fe		1.856	—	127	—	OER	Rajić et al. (2020)
ZnO@NMC	0.5 M KOH	−1.7 vs. Ag/AgCl	—	108	—	HER	Ubaidullah et al. (2020)
ZnO@NMC		0.82 vs. Ag/AgCl	—	318	—	OER	Ubaidullah et al. (2020)

The overall activity of the catalyst was evaluated by the potential difference (Δ*E*_OER/HER_) between the onset potential for OER and HER, where the smallest Δ*E*_OER/HER_ value indicates the better bifunctional electrode. Under light irradiation conditions, the smallest Δ*E*_OER/HER_ was obtained for 10ZnO@1RuO_2_-600 catalysts; the values of 2.34 V and 2.11 V in H_2_SO_4_ and NaOH, respectively, suggest their potential for bifunctional photoelectrode materials. In the dark condition, the same catalysts showed the smallest potential variation in HER/OER in the acidic solution, compared to 10ZnO@1RuO_2_ in the alkaline solution. The best bifunctional catalytic activity of 10ZnO@1RuO_2_-600 among all the evaluated catalysts can be explained by the synergy of the reduced number of bulk oxygen vacancies (Figure 8D) and the increased number of established heterojunctions (Figure 7), which can modulate the electronic structures, increase the number of active sites, and accelerate electron transfer (Liu et al., 2021).

The ORR activity of the catalysts was tested in both alkaline and acidic electrolytes. None of the tested catalysts showed good catalytic activity for ORR in the acidic electrolyte, while 10ZnO@1RuO_2_ showed the best activity for ORR in the alkaline electrolyte, with an onset potential of 0.633 V vs. RHE, Figure 9D. As the best catalyst for ORR in the alkaline electrolyte, 10ZnO@1RuO_2_ was additionally tested with a rotating disc electrode (RDE). Figure 9D shows the LSV curves of 10ZnO@1RuO_2_ at a rotation rate of 200–1,600 rpm and a scan rate of 5 mV⋅s^−1^. As shown in Figure 9E, the current density increases gradually with the rotation speed. However, the LSV curves at 800, 1,200, and 1,600 rpm overlap, probably because the total amount of oxygen in the electrolyte solution on the electrode surface was reduced at a rotation speed of 800 rpm when the maximum concentration gradient was reached; thus, further increases in rotation speed did not affect the current density (Rajić et al., 2020).

The mechanism of ORR in alkaline electrolytes can follow different pathways depending on the number of electrons; it can be a direct four-electron pathway (Eq. 6 or an indirect two-electron pathway (Eqs 7, 8) (Milikić et al., 2022):

$$O_{2}+2\;H_{2}O+4\;e^{-}\;\to \;4\;{OH}^{-}$$ (6)

$$O_{2}+H_{2}O+2\;e^{-}\;\to \;{HO}_{2}^{-}+{OH}^{-}$$ (7)

$${HO}_{2}^{-}+H_{2}O+2\;e^{-}\;\to \;3\;{OH}^{-}$$ (8)

Koutecky–Levich plots (*j* ^−1^ vs. ω^−1/2^) at different electrode potentials were constructed to calculate the electron transfer number (*n*) based on the Koutecky–Levich (K–L) equation (Chakrabarty et al., 2019). The Koutecky–Levich plots (Figure 9F) show parallel straight lines with nearly the same slope within the measured potential range, suggesting first-order reaction kinetics (Milikić et al., 2022). The *n* values calculated in the potential range between −0.75 and −0.60 V vs. RHE ranged from 2.59 to 2.24 electrons, pointing to two-electron pathways for the 10ZnO@1RuO_2_ composite as a catalyst. In the same potential range, the numbers of electrons were 3.75–4.13 for pristine ZnO and 1.89–1.65 for bare RuO_2_. The calculated *n* value indicated that when 10ZnO@1RuO_2_ composite was used as the ORR catalyst, molecular O_2_ was first reduced to HO_2_^−^ and then to H_2_O. The low ORR activity of the 10ZnO@1RuO_2_ composite with the less desirable two-electron pathway may be related to the neglected number of surface oxygen vacancies, as shown in this study by the PL spectra (inset in Figure 9D). Surface oxygen vacancies are well known to act as active sites for ORR and play an important role in the behavior of ORR catalysts (Fujii et al., 2015; Kan et al., 2016).

**FIGURE 10:**
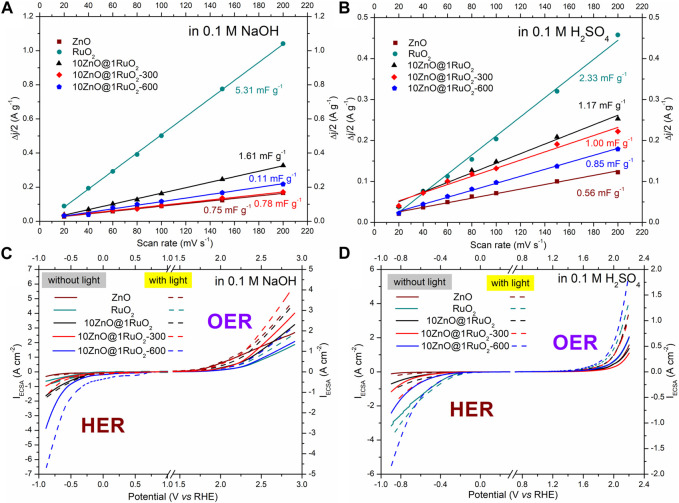
Calculated double-layer capacitance (Cdl) used to estimate the electrochemically active surface area of the samples in **(A)** 0.1 M NaOH and **(B)** 0.1 M H_2_SO_4_. ESCA-normalized LSV curves of the samples in 0.1 M NaOH **(C)** and 0.1 M H_2_SO_4_ **(D)**.

Finally, to determine the intrinsic HER and OER activity of the studied catalyst, the LSV data were normalized by the ECSA. The ECSA was estimated by recording CVs at scan rates of 20, 40, 60, 80, 100, 150, and 200 mV s^-1^ and using Eq. 5. The average current densities (*Δj* = (ja-jc)/2) were plotted as a function of the scan rate (Figures 10A, B), while *C*_dl_ was obtained as the slope of the linear fit (Perez Bakovic et al., 2021). The *C*_dl_ values of the studied samples are indicated in Figures 10A, B. The *C*_dl_ values of RuO_2_ in both electrolytes were higher than those of the other samples, indicating a larger ESCA, leading to better HER and OER activities, Figures 9A, B. The large ESCA of RuO_2_ is probably due to the significant agglomeration of RuO_2_ primary particles (Figures 5C, D). The ECSA-normalized LSV curves (Figures 10C, D) show that when the contribution of the larger ESCA of RuO_2_ is excluded, 10ZnO@1RuO_2_ -600 shows the best HER activity in both electrolytes and the best OER activity in the acidic electrolyte, while 10ZnO@1RuO_2_ -300 shows the best OER activity in the alkaline electrolyte. The best HER activity of 10ZnO@1RuO_2_-600 in both mediums as well as OER activity in the acidic medium, can be explained by the increased number of established heterojunctions, which increase the number of active sites, modulate the electronic structures, and accelerate electron transfer. The better OER activity of 10ZnO@1RuO_2_-300 in the alkaline medium can be explained by a larger number of surface oxygen vacancies, which induce more active sites and improve OH^−^ adsorption on the catalyst surface.

We previously showed that surface defects can improve the photoelectron-catalytic activity of ZnO-based materials while bulk defects, especially oxygen vacancies, can act as recombination centers, leading to a loss of photoelectron-catalytic activity (Marković et al., 2019). Thus, tuning the relative concentration ratio of surface to bulk defects in semiconductor nanocrystals can improve the photoelectro-catalytic efficiency of ZnO-based materials. Corby and co-workers showed that an intermediate number of defect states significantly reduced bulk recombination and improved electron transport, while high or very high defect concentrations resulted in a higher probability of trap-mediated recombination, thus lowering performance (Corby et al., 2020). Moreover, a bulk vacancy concentration of about 2% oxygen atoms was optimal for adequate charge separation and transport while minimizing bulk recombination through the generated defect states (Corby et al., 2020). Thus, even bulk oxygen vacancies induced intra-band defects, which were particularly important for charge separation and transport. Their influence on photoelectro-catalysis is a long-debated issue.

## 4 Conclusion

This study presented the results of the detailed physicochemical characterization of 10ZnO@1RuO_2_ composites and the potential for their application as bifunctional photo-electro catalysts for HER and OER in alkaline and acidic solutions. 10ZnO@1RuO_2_ composite nanoparticles were prepared by the microwave irradiation of Zn(OH)_2_ precipitated on commercial RuO_2_ particles in a 10:1 molar ratio. To determine the influence of annealing temperature on the photo-electro catalytic activity of the 10ZnO@1RuO_2_ composite, influential properties such as phase composition, crystallinity, Zn, Ru, and O elemental distribution, morphology, optical properties, and band gap structure were studied in detail. XRD and Raman spectroscopy revealed that additional annealing of 10ZnO@1RuO_2_ composite led to increased average crystallite size, shrinkage of both unit ZnO and RuO_2_ cells in the composite, and increased crystallinity with enhanced local (short-range) ordering in ZnO. The EDS mapping showed a homogenous distribution of the Zn, Ru, and O elements in the composite particles. UV-Vis DRS and PL spectroscopy revealed that a small amount of RuO_2_ in the 10ZnO@1RuO_2_ composite significantly increased the absorption in the visible region and reduced the number of bulk oxygen vacancies. The LSV studies showed that, compared to pristine ZnO, all investigated 10ZnO@1RuO_2_ composites showed enhanced catalytic activity toward HER in both alkaline and acidic solutions. Moreover, in the alkaline solution, 10ZnO@1RuO_2_-600 exhibited better activity toward HER than bare RuO_2_. 10ZnO@1RuO_2_-600 also showed the best catalytic activity toward OER in the acidic solution. In the alkaline solution, the as-prepared composite showed better catalytic activity toward OER than both annealed composites. The improved HER and OER activity of the composites compared to pristine ZnO can be explained by the synergy of 1) improved electron transfer due to the metallic conductivity i.e., the low resistivity of RuO_2_, 2) the reduced number of bulk oxygen vacancies, which were further reduced by the composite annealing, and 3) the increased number of established heterojunctions, which modulated the electronic structures, increased the number of active sites, and accelerated electron transfer. The photo-electro catalytic activities of the ZnO-based composites were significantly improved by the addition of a small amount of RuO_2_ nanoparticles, which absorbed almost 100% of visible light. The low activities of the 10ZnO@1RuO_2_ composites toward ORR were additional confirmation that ORR preferred catalysts with sufficient surface oxygen vacancies.

## Data Availability

The original contributions presented in the study are included in the article/Supplementary Material, further inquiries can be directed to the corresponding authors.
