# Supplementary material for: Enhancement of ZnO@RuO2 bifunctional photo-electro catalytic activity toward water splitting
Source: Front Chem. 2023 Apr 27;11:1173910. doi: 10.3389/fchem.2023.1173910 (PMC10174304; doi:10.3389/fchem.2023.1173910)
Supplement: Supplementary file 1 [file DataSheet1.docx]

Supplementary Material

Enhancement of ZnO@RuO_2_ bifunctional photo-electro catalytic activity toward water splitting

Katarina Aleksić^1†^, Ivana Stojković Simatović^2†*^, Ana Stanković^1^, Ljiljana Veselinović^1^, Stevan Stojadinović^3^, Vladislav Rac^4^, Nadežda Radmilović^5^, Vladimir Rajić^5^, Srečo Davor Škapin^6^, Smilja Marković^1†*^

^1^Institute of Technical Sciences of SASA, Belgrade, Serbia

^2^Faculty of Physical Chemistry, University of Belgrade, Belgrade, Serbia

^3^Faculty of Physics, University of Belgrade, Belgrade, Serbia

^4^Faculty of Agriculture, University of Belgrade, Zemun, Serbia

^5^Vinča Institute of Nuclear Sciences, University of Belgrade, Belgrade, Serbia

^6^Jožef Stefan Institute, Ljubljana, Slovenia

*** Correspondence:** Ivana Stojković Simatović and Smilja Marković
ivana@ffh.bg.ac.rs, and [smilja.markovic@itn.sanu.ac.rs](mailto:smilja.markovic@itn.sanu.ac.rs)

# Supplementary Figures


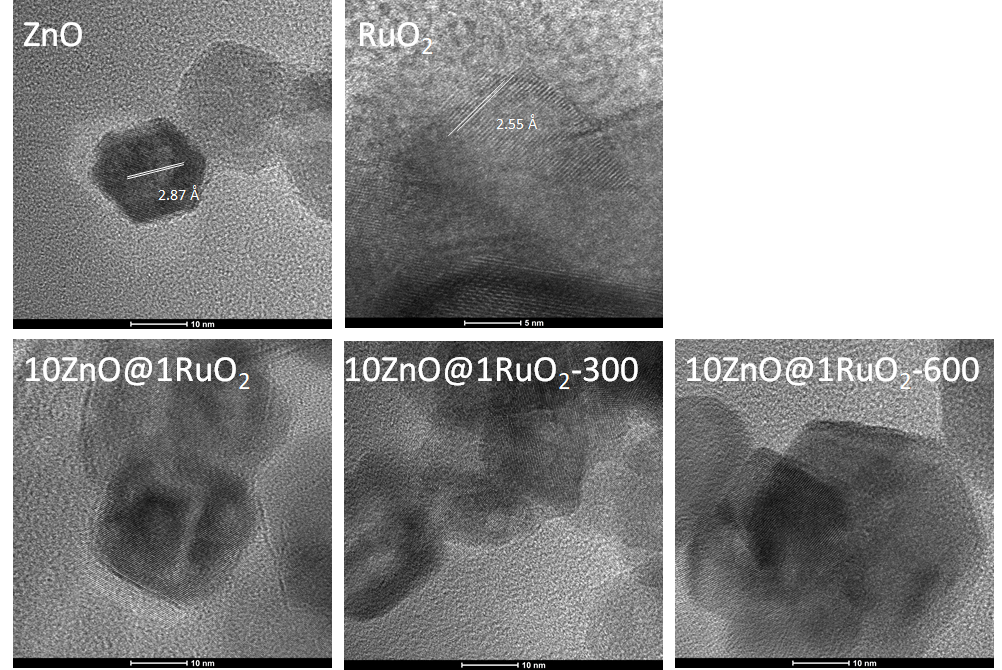


**Supplementary Figure 1.** HRTEM images of the pristine ZnO, bare RuO_2_, 10ZnO@1RuO_2_, 10ZnO@1RuO_2_-300, and 10ZnO@1RuO_2_-600 particles.

**
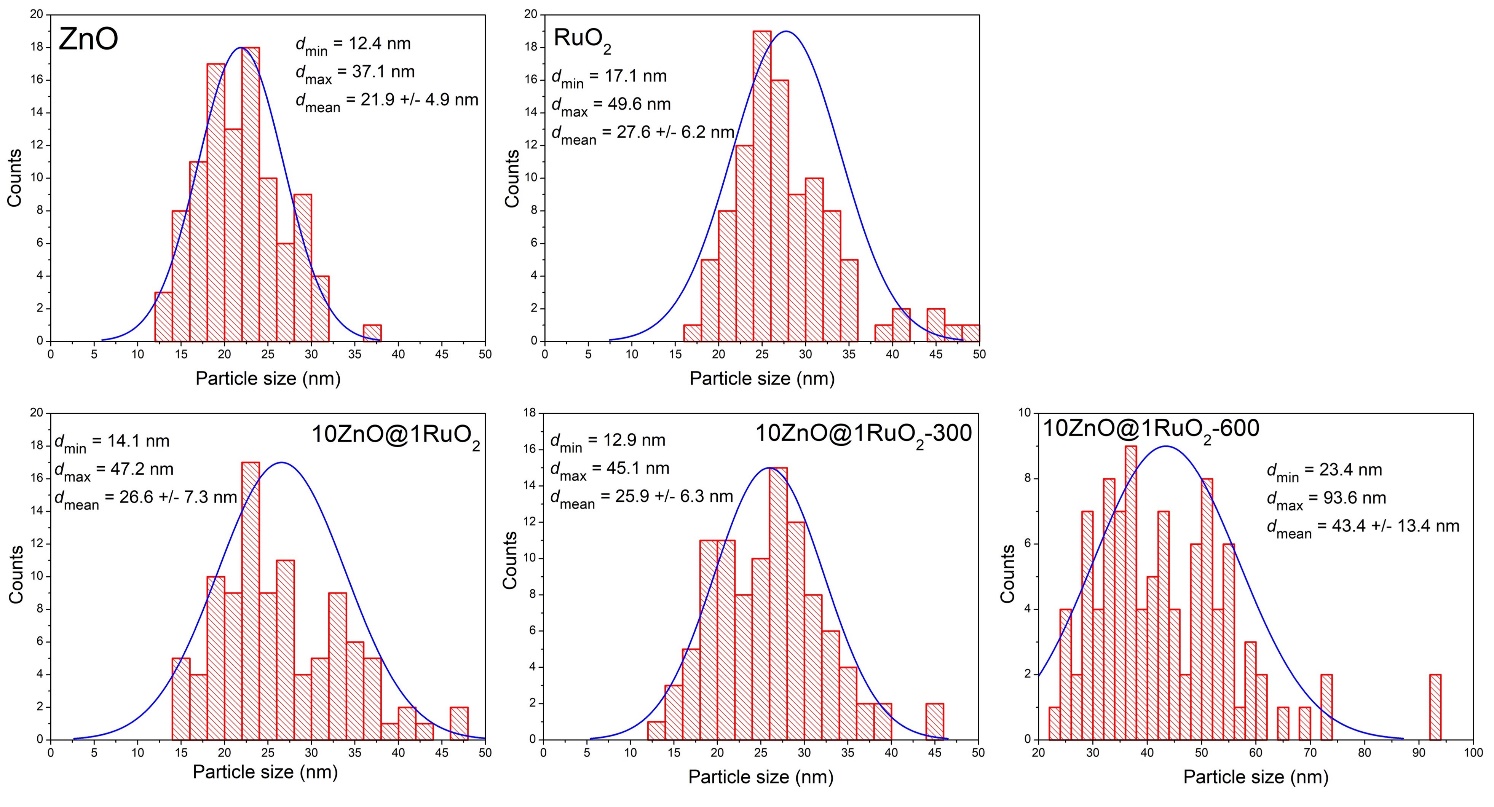
**

**Supplementary Figure 2.** Particle size distribution histograms estimated from TEM images.
